# Oxidative Stress and Antioxidants in Atherosclerosis Development and Treatment

**DOI:** 10.3390/biology9030060

**Published:** 2020-03-21

**Authors:** Anastasia V. Poznyak, Andrey V. Grechko, Varvara A. Orekhova, Yegor S. Chegodaev, Wei-Kai Wu, Alexander N. Orekhov

**Affiliations:** 1 Institute for Atherosclerosis Research, Skolkovo Innovative Center, 121609 Moscow, Russia; tehhy_85@mail.ru; 2 Federal Research and Clinical Center of Intensive Care Medicine and Rehabilitology, 109240 Moscow, Russia; noo@fnkcrr.ru; 3 Laboratory of Angiopathology, Institute of General Pathology and Pathophysiology, 125315 Moscow, Russia; varvaraao@gmail.com; 4 I. M. Sechenov First Moscow State Medical University (Sechenov University), 119146 Moscow, Russia; egozavr-ch@mail.ru; 5 Department of Internal Medicine, National Taiwan University Hospital, Bei-Hu Branch, Taipei 100, Taiwan; weikaiwu0115@gmail.com; 6 Institute of Human Morphology, 117418 Moscow, Russia

**Keywords:** atherosclerosis, oxidative stress, NADPH oxidase

## Abstract

Atherosclerosis can be regarded as chronic inflammatory disease affecting the arterial wall. Despite the recent progress in studying the pathogenesis of atherosclerosis, some of the pathogenic mechanisms remain to be fully understood. Among these mechanisms is oxidative stress, which is closely linked to foam cells formation and other key events in atherosclerosis development. Two groups of enzymes are involved in the emergence of oxidative stress: Pro-oxidant (including NADPH oxidases, xanthine oxidases, and endothelial nitric oxide synthase) and antioxidant (such as superoxide dismutase, catalases, and thioredoxins). Pro-oxidant enzymes in normal conditions produce moderate concentrations of reactive oxidant species that play an important role in cell functioning and can be fully utilized by antioxidant enzymes. Under pathological conditions, activities of both pro-oxidant and antioxidant enzymes can be modified by numerous factors that can be relevant for developing novel therapies. Recent studies have explored potential therapeutic properties of antioxidant molecules that are capable to eliminate oxidative damage. However, the results of these studies remain controversial. Other perspective approach is to inhibit the activity of pro-oxidant enzymes and thus to slow down the progression of atherosclerosis. In this review we summarized the current knowledge on oxidative stress in atherosclerosis and potential antioxidant approaches. We discuss several important antioxidant molecules of plant origin that appear to be promising for treatment of atherosclerosis.

## 1. Introduction

Oxidative stress was first defined in 1985 by Helmut Sies as “a disturbance in the pro-oxidant to antioxidant balance in favor of the oxidant species, leading to potential damage”. Introduction of this concept resulted in the development of a new research area named Redox Biology [1,2]. The modern concept of oxidative stress includes more details: “the lack of balance between the occurrence of reactive oxygen/nitrogen species (ROS/RNS) and the capacity of organism to counteract their action by the antioxidative protection systems” [3]. ROS include both free radical and non-free radical chemically active compounds that contain oxygen, among them hydrogen peroxide (H_2_O_2_), superoxide (O_2_∙), singlet oxygen (1/2 O_2_), and the hydroxyl radical (∙OH). Reactive nitrogen, iron, copper, and sulfur species have also been described [4]. Under physiological conditions, ROS and RNS are produced at moderate concentrations and play an important role in cell signaling, regulation of cell cycle, apoptosis and gene expression through interaction with transcription factors. Moreover, ROS are generated by phagocytes that use them to kill pathogens and combat infection [5,6]. Intense physical activity and exposure to toxins can also lead to increased ROS and RNS production.

Low levels of superoxide are generated as a byproduct of mitochondrial oxidative phosphorylation and are converted to hydrogen peroxide and subsequently to water [7]. Under pathological conditions, mitochondrial oxidative stress can occur due to excessive ROS production or failure of antioxidant mechanisms. Atherosclerosis is one of the common human pathologies, which is known to be associated with mitochondrial oxidative stress. The important role of oxidative stress in the disease development has also been confirmed in animal models of atherosclerosis [8,9].

According to the current understanding, oxidative stress occurs due to increased production of ROS/RNS and/or insufficient antioxidant protection [3]. Damage incurred by free radicals is a well-known contributor to many human chronic pathologies, such as cancer, emphysema, cataract, neurodegenerative disorders, and inflammatory and cardiovascular diseases [2,10]. It was roughly estimated that over 100 disorders are associated with oxidative stress, in many of them playing a causative or disease modifying role. ROS also cause an irreversible progression of oxidative decay having negative impact on physiological functions, promoting disease incidence, and reducing the life span [10]. Correspondingly, antioxidants have long been considered as attractive potential therapies for many human diseases. However, numerous clinical studies failed to demonstrate the beneficial effects of antioxidant supplements on the target pathologies. For example, the protective effect of vitamin E for prevention of cancer and major cardiovascular outcomes could not be proven [11].

## 2. Oxidative Stress in Atherosclerosis

Oxidative stress is a well-known component of atherosclerosis pathogenesis, occurring in parallel with activation of pro-inflammatory signaling pathways and expression of cytokines/chemokines [12]. Most of the identified risk factors for atherosclerotic cardiovascular disease (CVD), including dyslipidemia, diabetes, and hypertension, are accompanied with increased ROS production in the vessel wall [13,14]. Disturbance of the endothelial function is considered to be the first key event in atherogenesis development, which breaches the balance between vasoconstriction and vasodilatation, increases the endothelial permeability and triggers a local inflammatory response. It results in the infiltration of inflammatory cells from circulation into the vessel wall and indirect induction of cytokines and other inflammatory mediators [15].

Low-density lipoprotein (LDL) is the major source of lipids accumulation in atherosclerotic plaques. However, to become atherogenic, LDL needs to undergo certain chemical modifications [16]. LDL particles undergo numerous modifications in blood plasma [17]. The most studied of these modifications are probably desialylition and oxidation. Desialylated LDL particles were also shown to contain less antioxidants and dwell for a longer time in the subendothelial space of the arterial wall, which may explain their increased susceptibility to oxidation. As a result, atherogenic multiple-modified LDL is generated in atherosclerosis-prone individuals [18,19]. Oxidized LDL accumulates in predisposed for atherosclerosis areas of the arterial wall and contributes to the increased expression of vascular cell adhesion molecules-1 (VCAM-1), P, and E- selectins and other cell adhesion molecules on the endothelial cells. Activation of the endothelial VCAM-1 expression is driven by a range of inflammatory signals and is performed through an antioxidant-inhibitable mechanism, which involves a redox-sensitive activation of nuclear transcription factor NF-kB [20]. Enhanced expression of cell adhesion molecules results in leukocyte recruitment and infiltration into the sub-endothelial space of the arterial wall. Once inside the intimal layer, monocytes differentiate into macrophages and internalize modified lipoproteins becoming foam cells. Presence of foam cells in the arterial wall is a hallmark of early atherosclerotic lesion [21]. 

Oxidative stress plays an important role in atherosclerotic lesion formation due to ROS overproduction. Endothelial cells and smooth muscle cells are capable of producing oxidants through the activity of several enzymes. The bulk amount of ROS in the vascular wall is produced by a membrane-associated group of enzymes typical for cells of mesodermal origin called NADPH oxidases [22]. Apolipoprotein E knock-out (*apoE^-/-^*) mice that are also heterozygous for superoxide dismutase (SOD2^+/−^) knockout are characterized by accelerated atherosclerosis development in the arterial branch points [8]. Animal models have also shown that atherosclerotic processes may be reversed under certain conditions, therefore providing the opportunity for therapy development [9].

In parallel to monocytes/macrophages migration into the intima, recruitment of mast cells and T-lymphocytes also takes place. The increased presence of the immune cells in the arterial wall leads to cytokines release and the induction of the inflammatory processes and ROS production. For instance, TNF-α was shown to increase mitochondrial ROS production; IL-1β—to induce ROS production by NADPH oxidase; and IFN-γ—to induce ROS through both mitochondrial and NADPH oxidase pathways [23,24]. Therefore, atherosclerosis plaque development is a result of the production and release of both growth factors and ROS. Moreover, ROS can enhance the expression of scavenger receptors on vascular smooth muscle cells therefore inducing their ability to internalize and accumulate lipids and transform into foam cells. The release of matrix metalloproteinases (MMPs), which is responsible for plaque disruption, is also stimulated by ROS. Cyclic strain-induced MMP-2 expression on vascular smooth muscle cells was shown to be dependent on NADPH oxidase activation [25].

## 3. Main ROS-Producing Enzymes in Atherosclerosis

Several ROS-producing systems are present in the vascular wall, including NADPH oxidases, xanthine oxidase, enzymes of the mitochondrial respiratory chain, and a dysfunctional, uncoupled endothelial NO synthase (eNOS) [13,26]. Noteworthy, mechanisms of cross-regulation exist between these pro-oxidant systems. According to the theory of “kindling-bonfire radicals”, it is possible to classify ROS sources into two groups: initial (e.g., mitochondrial respiratory chain and NADPH oxidases) and secondary (e.g., xanthine oxidase, uncoupled eNOS). The crosstalk between the members of these two groups of enzymes occurs through ROS generation: ROS produced by the initial source trigger the activation of secondary sources [27]. Therefore, the “kindling radicals” theory postulates that the primary, NADH oxidase-derived ROS, are kindling the production of ROS by secondary sources and, subsequently, a tertiary source, which is believed to be the mitochondria. This results in a “bonfire” of radicals and oxidative stress [27]. The resulting cascade of ROS production has been identified as inflammasome activator and an important mechanism underlying human inflammatory disorders [28].

### 3.1. NADPH Oxidases

NADPH oxidases are multisubunit enzyme complexes that produce superoxide from molecular oxygen using NADPH as the electron donor. NADH oxidases consist of two membrane-bound subunits, p22phox and a Nox homologue, and several cytosolic regulatory subunits [29]. Initially, Nox was found to be expressed in the membrane of “professional” phagocytic cells of the immune system that generate relatively large amounts of ROS to facilitate killing of the pathogens [30]. Presence of NADPH oxidase homologues was further revealed in nonphagocytic cells, such as the endothelial cells (ECs) and smooth muscular cells (SMCs) [31]. Seven types of Nox have been identified in humans: Nox1-5, Duox1 and Duox2. Among them, only Nox1, Nox2, Nox4, and Nox5 are expressed in the endothelium, vascular SMCs, fibroblasts, or perivascular adipocytes at significant levels. Accumulating evidence demonstrates that Nox homologues play important and various roles in atherogenesis [32]. 

Nox1 and Nox2 were shown to be pro-atherogenic. Genetic deletion of Nox1 in *apoE^-/-^* mice led to alleviation of atherosclerosis. Deficiency of Nox2 diminished atherosclerosis in the descending aorta of model animals but did not alleviate the pathology progression in the aortic sinus [33]. By contrast, Nox4 demonstrated an anti-atherosclerotic effect in murine models [34,35]. Nox5 gene is absent from the rodent genome and is therefore the most challenging homologue to study. Enhanced expression of Nox5 was observed in human atherosclerotic lesions [36]. It was also found to be associated with human hypertension [37] and diabetic nephropathy [38]. Transgenic mice with the podocyte-specific expression of human Nox5 in the kidneys (Nox5βpod^+^ mice) exhibited early onset of renal dysfunction, including albuminuria, podocyte effacement, glomerular basement membrane thickening, interstitial fibrosis, and elevated systolic blood pressure [38]. Together, these findings indicate that Nox5 may also be involved in atherogenesis through an unknown mechanism.

### 3.2. Xanthine Oxidases

Xanthine oxidases use molecular oxygen as electron acceptor to generate hydrogen peroxide and superoxide anions [39]. These enzymes are normally present in the ECs and in blood plasma, and their levels in atherosclerotic plaques were found to be increased [40]. Several studies highlighted the possible involvement of xanthine oxidases in atherosclerosis development. Both expression and activity of endothelial xanthine oxidases could be increased by oscillatory shear stress and treatment with angiotensin II [41,42]. It was shown that atherogenesis in *apoE^-/-^* mice could be reduced by xanthine oxidase inhibitors [43]. Moreover, inhibition of xanthine oxidase allowed reducing the endothelial dysfunction in heavy smokers [44]. Xanthine oxidases stimulate the expression of LOX-1 and CD-36 in macrophages and vascular smooth muscle cells. Interestingly, xanthine oxidases generate uric acid, high blood concentration of which can lead to clinical manifestation of gout, which is associated with increased incidence of atherosclerosis-related events. Elevated level of uric acid also triggers foam cell formation through stimulating the expression of important scavenger receptor CD-36 that is responsible for the binding and uptake of oxidized LDL in macrophages [45].

### 3.3. Uncoupled Endothelial Nitric Oxide Synthase

Under normal conditions, eNOS produces nitric oxide (NO) that acts as a major vasoprotective factor of the endothelium. However, functioning of the eNOS may be disrupted in human pathologies associated with oxidative stress [46]. Rapid oxidative inactivation of NO by the excess superoxide links the oxidative stress to the endothelial dysfunction. Persistent oxidative stress renders eNOS uncoupled (uncoupling of O_2_ reduction from the NO synthesis) resulting in the production of superoxide instead of NO. The likely causes of eNOS uncoupling include the deficiency of eNOS cofactor tetrahydrobiopterin (BH4), deficiency of eNOS substrate l-arginine, and eNOS S-glutathionylation. Peroxinitrite is a direct product of reaction between NO and superoxide that can cause BH4 deficiency due to the ability to oxidize BH4 [47]. In *apoE^-/-^* mice, enhanced oxidative degradation of BH4 and eNOS uncoupling could be seen in cardiovascular tissues [48]. Evidence of ROS production by uncoupled eNOS has been obtained in patients with atherosclerosis [49], as well as in subjects with hypercholesterolemia [50], hypertension [51], diabetes mellitus [52], and in chronic smokers [53].

## 4. Antioxidant Enzymatic Systems

Antioxidants are molecules that have the ability to inhibit oxidation of other molecules, which is realized through scavenging oxidants or decreasing the production of ROS. Physiologically, antioxidants prevent damage from oxidants and free radicals, but do not block redox reactions required for metabolism, energy production, signaling, and other cellular function [54]. Antioxidant molecules are highly oxidizable compounds that react quickly with free radicals forming stable derivatives or relatively stable free radicals. Based on their origins, small molecule antioxidants can be endogenous (e.g., uric acid, coenzyme Q, bilirubin) or exogenous (e.g., vitamins C and E, flavonoid, carotenes) [55,56]. Major antioxidant systems that are present in the vascular wall include superoxide dismutases, catalase, glutathione peroxidase, paro-oxanase, and NO synthases [13].

### 4.1. Superoxide Dismutases

Superoxide dismutases (SODs) interact with superoxide transforming it to hydrogen peroxide that undergoes further enzymatic processing by glutathione peroxidases, catalases, and thioredoxins [46]. Three isoforms of SODs have been described: SOD1 is found in the cytoplasm and on the inner mitochondrial membrane; SOD2 is located in the mitochondrial matrix, and SOD3 is extracellular. Upregulation of SODs does not lead to the reduction of ROS production directly, because of the high amounts of distal oxidants produced from hydrogen peroxide, that also contribute to the atherosclerosis development. In *apoE^-/-^* mice, overexpression of both SOD1 and catalase, which can degrade hydrogen peroxide, reduced atherosclerosis, while overexpression of SOD1 alone, on the contrary, could enhance the pathology development [57]. It may be concluded that SODs have a bidirectional effect: Although they are able to inhibit anion-mediated damage, they can trigger oxidative stress in the absence of sufficient downstream enzymes that detoxify SODs end products [13].

### 4.2. Catalases

These enzymes decompose hydrogen peroxide to water and oxygen. Within the cell, catalases are found in the peroxisomes. It was demonstrated that catalases may improve atherosclerosis in in high-fat diet mice models [57]. Studies in *apoE^-/-^* mice showed that decrease of aortic content of F2-isoprostanes and alleviation of atherosclerosis occurred in response to overexpression of catalases in combination with SOD-1. However, the mechanisms of such atheroprotective effect remain unclear [57,58]. The enhanced activity of catalases was revealed in foam cells obtained from atherosclerotic lesions from rabbit aortas [59]. EUK-8, a synthetic compound that mimics SOD and catalase activities at once, was reported to prevent remodeling of the left ventricle and cardiac decompensation in mice model developing heart failure [60].

### 4.3. Thioredoxins

Thioredoxin system consists of both thioredoxin (Trx) and thioredoxin reductase (TrxR) that can reduce hydrogen peroxide and target proteins. The two mammalian isoforms if Trx (Trx1 and Trx2) and three izoenzymes of TrxR (Trx1-3) are broadly expressed and can be found intra- and extracellularly. The function of oxidant scavenging is implemented through the Trx peroxidases or peroxiredoxins (Prx) [61]. Under conditions of chronic laminar shear stress in an experimental cellular model, Trx-1 and Prx-1 were shown to be overexpressed in bovine aortic ECs and to protect them from oxidative stress-related damage [62]. Expression of Trx was also observed in medial SMCs of coronary arteries in healthy humans, and the expression pattern broadened in atherosclerotic arteries, involving the whole arterial wall and macrophages [63].

The involvement of Trx system in atherogenesis at different stages was further confirmed by clinical and experimental studies. The system was shown to play an important role in the regulation of metabolic processes, insulin signaling, regulation of blood pressure and inflammation. Levels of plasma Trx were found to be increased in patients with atherosthrombosis. Together these findings highlight the Trx system as a promising point of therapeutic intervention for treatment of such diseases as atherosclerosis, diabetes and metabolic syndrome [64]. Downregulation of cytosolic isoform of thioredoxin, Trx1, was shown to inhibit the expression of VCAM-1 and ICAM-1 and prevent atherosclerosis initiation of [65]. Prothrombotic phenotype in mouse models together with the endothelial dysfunction could be caused by the suppression of the mitochondrial isoform of thioredoxin, Trx2 [66]. Inflammatory and chemotactic activity of macrophages may also be regulated, at least in part, via modulation of their phenotypes at different stages of atherosclerotic plaque formation by the Trx system [64]. Since TrxR is a selenoenzyme, it may be involved in conveying the beneficial effects of selenium supplementation on atherosclerosis development [67]. 

### 4.4. Glutathione Peroxidases

Glutathione peroxidases (GPXs) is another family of selenoproteins that are responsible for controlling the oxidative stress, especially during inflammation. In humans, 4 isoforms of GPXs are known, each with the selenocysteine at the active site. Increased proliferation of vascular SMCs at the atherosclerotic lesion site can be enhanced by oxidative stress. A role of GPX-1 in normalization of vascular SMC proliferation rate via inhibition of matrix metalloproteinase 9 (MMP9) and in restoring the endothelial function has been reported [68]. The possible protective role of GPX in atherosclerosis GPX deficiency in mouse peritoneal macrophages was shown to increase oxidized LDL-induced foam cell formation and macrophages proliferation. Cheng and colleagues demonstrated that overexpression of GPX-1 in *apoE^-/-^* mice inhibited the expression of adhesion molecules by the ECs and monocytes, as well as and other pro-atherogenic processes [69]. These findings support the likely protective role of GPXs in atherosclerosis and may contribute to the development of future therapies.

### 4.5. Paraoxonases

Paraoxonases (PON) is a family of enzymes, comprising three isoforms: PON1, PON2, and PON3. These enzymes were shown to reduce the oxidative stress, lower lipid peroxidation, and reduce atherosclerosis in animal models [13]. PON1 is associated with high-density lipoprotein (HDL), and its overexpression has a protective effect against atherosclerosis in *apoE^-/-^* mice [70]. PON2 is expressed by the vascular wall cells. Inside the cell, it resides in the membranes of the ER and mitochondria. PON2 has the ability to decrease the superoxide formation. It was shown to translocate to the plasma membrane in response to lipid peroxidation and to decrease oxidative stress in mice models [71]. Expression of PON3 was found to be low in vascular SMCs from atherosclerotic plaques suggesting for its protective role. Moreover, PON3 could inhibit mitochondrial superoxide formation [72]. 

### 4.6. Nitric Oxide Synthases

Nitric oxide synthases (NOS) play either anti- or pro-oxidant roles in atherosclerosis. NO produced by endothelial nitric oxide (eNOS) that is constitutively expressed in the endothelial cells inhibits LDL oxidation, leukocyte adhesion and migration, vascular SMC proliferation, and platelet aggregation [73]. Deletion of eNOS enhanced atherosclerosis in *apoE^-/-^* mice [74]. Neuronal NO synthases are constitutively expressed in central and peripheral nerve cells, but also can be found in the vascular wall, which indicates for possible antiatherogenic role of nNOS. Inducible NOS (iNOS), is activated during inflammation, sepsis and oxidative stress and acts in a pro-atherogenic way. Formation of a pro-atherosclerotic oxidant peroxynitrite by iNOS is likely to play a key role in conveying the pro-atherogenic effect of iNOS [75]. Moreover, activation of iNOS may result in relative deficiency of BH4 for eNOS, thereby uncoupling eNOS, and forming a major source of ROS that contributes to atherogenesis.

## 5. Challenges in the Development of Therapeutic Strategies Targeting Oxidative Stress

Due to the significant impact of oxidative stress on atherosclerosis initiation and development, many recent studies were focused on identifying effective therapeutic approaches based on antioxidant effects of various compounds [76]. However, none of the tested antioxidants could form the basis of anti-atherosclerosis therapy. The efficacy of vitamins C and E and folic acid against atherosclerosis could not be demonstrated in clinical studies. Possible reasons of this failure include the differences in oxidative stress in patients that were not assessed for their “oxidative stress status” [77]. Moreover, the mode and duration of vitamin administration, its combination with other potential antioxidant drugs, and the lack of specificity of action may have also influenced the result of trials [78]. Therapies that are commonly used for treatment of atherosclerosis, such as statins, angiotensin receptor AT1 antagonists and angiotensin converting enzyme (ACE) inhibitors, often show pleiotropic antioxidant effects [79]. For instance, statins were observed to inhibit NADPH oxidase activation and to show antioxidant properties in preclinical models [80]. Therefore, the search for anti-oxidant approaches that could be effective against atherosclerosis currently continues. The focus is shifted from systemic antioxidants, such as vitamins C and E to more targeted therapies that can tackle local oxidative stress at the site of atherogenesis. Another important direction of research is the search for natural compounds that can be used for prolonged treatment without causing significant adverse effects. Numerous natural compounds were found to have antioxidant and anti-inflammatory properties that may be beneficial for the further atherosclerosis treatment approaches development. The antioxidant precursor of glutathione, N-acetylcysteine, and polyphenols appear to be promising potential therapeutic agents, along with food products and dietary supplements that contain these substances, such as black and green tea, red wine, and extra virgin olive oil [81]. Antiatherogenic properties of N-acetylcysteine, polyphenols, flavonoid conjugates and isoquercetine remain to be tested. However, promising results have been reported for isoflavonoid-rich herbal preparations [82].

### 5.1. Resveratrol

One promising compound with potential anti-atherosclerotic activity is resveratrol, a polyphenolic compound, which is present in many edible plants. Considerable amounts of dietary resveratrol are coming from the consumption of fruits, berries and wine. In in vitro systems resveratrol was shown to directly scavenge peroxynitrite, hydroxyl radical and other oxidants. However, in vivo studies revealed that the most important effect of resveratrol is on gene level [83]. Whole-genome microarray experiments on mice kept on high-calorie diet revealed changes of the expression patterns of 782 out of 41,534 individual genes in response to resveratrol treatment [84]. Preclinical studies of resveratrol identified numerous biological effects induced by this compound that could be explained by the variety of its molecular targets. Among them the most significant for the antioxidant effect were SIRT1 (NAD^+^-dependent histone/protein deacetylase sirtuin 1) [85], Nrf2 (nuclear factor-E2-related factor-2) [86] and ER (estrogen receptor) [87]. These results indicate that resveratrol is more than just antioxidant but also a powerful gene regulator.

### 5.2. Quercetin

Quercetin is a flavonoid compound that shows anti-inflammatory and antioxidant properties and also affects lipid metabolism. All these activities are beneficial in scope of atherosclerosis treatment. Enhanced reversed cholesterol transport was shown in *apoE^-/-^* mice fed a high-fat diet in response to the quercetin treatment [88]. A decrease in the production of inflammatory mediators, such as COX, 5-LOX, MPO, NOS, and CRP, was observed in hypercholesterolemic diet-fed atherosclerotic rats after quercetin supplementation [89]. Both these effects resulted in the retardation of atherosclerotic development. Antioxidant properties of quercetin in atherosclerosis is believed to be implemented through the upregulation of the autophagy in endothelial cells [90,91].

### 5.3. Melatonin

N-acetyl-5-methoxytryptamine (melatonin) was reported to positively affect cardiovascular diseases, including myocardial ischemia-reperfusion, hypertension, and heart failure. Being the main predominant indoleamine of the pineal gland, melatonin shows significant antioxidant properties [92]. Study by Ma et al. revealed that melatonin can promote ROS scavenging by mitophagy in macrophages within and thus to suppress prolonged NLRP3 inflammasome activation in atherosclerotic lesions, which resulted in attenuation of atherosclerosis progression in *apoE^-/-^* mice fed a high-fat diet [93].

### 5.4. Curcumin

Curcumin is a dietary pigment obtained from turmeric that has attracted much attention because of its multiple biological activities. Among all, the compound has demonstrated an antioxidant potential. Curcumin was shown to lower the expression of pro-atherogenic cytokines including MCP-1, IL-1β, and TNF-α in primary human monocytes and to induce anti-inflammatory M2 polarization of murine macrophages [94]. Atherosclerotic lesions in *apoE^-/-^* mice and fatty streaks in high-fat fed rabbits were smaller after treatment of animals with curcumin [95,96]. In randomized double-blind trial curcumin appeared to lower the cardiovascular risk in individuals with type 2 diabetes [97].

### 5.5. Other Potential Anti-Oxidant Agents

Flavonoids stand out as promising anti-oxidant agents that may prove to be beneficial for treatment of human diseases associated with chronic inflammation, including atherosclerosis. For instance, flavonoid-rich fraction of bergamot juice was shown to reduce lipopolysaccharides-induced inflammation in rats [98], and flavonoid-rich extract of orange juice was observed to reduce oxidative stress in an experimental model of inflammatory bowel disease [99]. These findings reflect the capacity of flavonoids to positively affect the oxidative stress and related inflammation. Another promising natural compound with anti-inflammatory properties is palmitoylethanolamide [100]. Palmitoylethanolamide in combination with polydatin, a natural precursor of resveratrol, showed an anti-inflammatory effect and also reduced vascular damage and expression of adhesion and inflammatory molecules, such as VCAM, ICAM-1, TNF-α, and IL-1β [101]. Anti-oxidant activities of foods continue to be revealed in animal studies. For instance, pistachios, both raw natural and roasted salted, were shown to reduce the production of pro-inflammatory cytokines and adhesion molecules and to reduce myocardial tissue injury in rats [102]. Future studies will help revealing the active anti-oxidant components of natural preparations and functional foods that will be useful for atherosclerosis prevention and treatment. However, the complexity of redox mechanisms within living tissues, such as arterial wall, make it challenging to develop an integrated analytical method to directly evaluate the antioxidant activity of various compounds. Standardization of analytical methods is an important step in that direction. Next, combination of several assays, such as measurement of ROS/RNS levels combined with a cellular assay to assess the cellular antioxidant response may yield good results [6].

## 6. Conclusions

It is now clear that oxidative stress is a more complicated process then simple damage induced by free radicals. A range of enzymatic systems, both pro-oxidant and antioxidant, are involved in oxidative stress development, with some of the key enzymes well-characterized to date. However, some of the well-known antioxidants, such as vitamins E and C, failed to demonstrate efficacy against the oxidative stress associated with atherosclerosis in clinical trials, indicating that more specific antioxidant drugs are needed. Further investigation of currently existing drugs may also reveal their antioxidant efficacy. Improvement of the assays measuring the antioxidant activity and combination of several analytical approaches is another important task of the future research.
